# Bioprospecting of Plant-Beneficial *Bacillus* Species for Growth Promotion and Disease Suppression

**DOI:** 10.21315/tlsr2025.36.2.1

**Published:** 2025-07-31

**Authors:** Ruth Meike Jayanti, Ike Marisna, Jilan Tsani Abdullah, Suryanti, Tri Joko

**Affiliations:** 1 Department of Plant Protection, Faculty of Agriculture, Universitas Gadjah Mada, Flora Street No. 1, Bulaksumur, Yogyakarta 55281, Indonesia; 2 Faculty of Agriculture and Business, Satya Wacana Christian University, Diponegoro Street No. 52–60, Salatiga 50711, Indonesia

**Keywords:** *Bacillus*, PGPB, *gyrB*, Shallot, Twisted Disease

## Abstract

Plant growth-promoting bacteria (PGPB) have been reported to promote plant growth and protect against plant diseases effectively. PGPB can control plant diseases through direct and indirect mechanisms. The direct mechanism involves the ability to provide nutrients and phytohormones. In contrast, the indirect mechanism refers to the ability to suppress the activity of pathogens through the production of various compounds and metabolites. The purpose of this study is to evaluate the plant health-promoting potential of *Bacillus* species. Several genetic determinants in 18 isolates of PGPB were investigated via polymerase chain reaction based on the genes *fenD, sfp, bamC, ituA, aiiA, ipdC and nifH*. Plant-beneficial traits were confirmed through seedling growth tests and *in vitro* antagonistic assays in the laboratory, followed by a field experiment that used selected *Bacillus* isolates to improve plant growth and control twisted disease in shallots. Results revealed that two *Bacillus* isolates, B-27 and RC76, have potential as PGPB. Isolates B-27 and RC76 were identified as *Bacillus velezensis* and *B. tropicus*, respectively, based on *gyrB* sequence analysis. The application of *B. velezensis* B-27 by spraying resulted in the lowest intensity of twisted disease in shallots. In addition, combined treatment with *B. velezensis* B-27 and *B. tropicus* RC76 increased plant height and leaf number.

**Highlights**

- 18 isolates of PGPB (B-27, EP3, RC76, EA64, Tlg4, Dm2, RB77, EB62, A8, A9, A10, A11, KP A003, KP A004, KP B51, Klt D04, Brb T1, Brb B01) were investigated based on plant-beneficial traits via PCR detection, seedling growth test, *in vitro* antagonistic assay and field experiment.
- Two selected *Bacillus* isolates, B-27 and RC76, with high potential as PGPB, were identified as *Bacillus velezensis* and *Bacillus tropicu*s, respectively, based on *gyrB* sequence analysis.
- The application of *B. velezensis* B-27 could suppress disease development of twisted disease in shallot. Meanwhile, the application of combined *B. velezensis* B-27 and *B. tropicus* RC76 increased plant height and leaf number.

## INTRODUCTION

Plant growth-promoting bacteria (PGPB) include free-living bacteria that form specific symbiotic relationships with plants, endophytic bacteria that can partially colonise plant tissues (Glick 2012), and bacteria in the rhizosphere or on root surfaces (Navitasari *et al*. 2020). PGPB can promote plant growth and protect plants from diseases and abiotic stresses through direct and indirect mechanisms. The direct mechanism includes bacterial activities that affect plant growth directly. It also includes auxin, 1-aminocyclopropane-1-carboxylate (ACC) deaminase, cytokinin and gibberellin production, nitrogen fixation, phosphate solubilisation and iron bond formation by siderophore bacteria. Indirect mechanisms refer to inhibiting one or more plant pathogens, including fungi and bacteria. Indirect mechanisms include antibiotic and cell wall-degrading enzyme production, competition, systemic resistance (ISR) induction, quorum quenching and siderophore production (Olanrewaju *et al.* 2017).

Many *Bacillus* species can act as PGPB since they are reported to produce a wide variety of secondary metabolites. Secondary metabolite production and antimicrobial activity are determinants of the ability of *Bacillus* species to control diseases. Secondary metabolites can be antibiotics from the surfactin group (surfactin and lichenysins), the iturin group (iturins A, C, D and E; bacyllomicins D, F and L and mycosubtilin), and the fengycin group (fengycins and plastatin) as well as amyl polyols, such as zwittermicin A (Stanković *et al.* 2012). The beneficial traits of *Bacillus* as a biocontrol agent and plant growth promoter include the ability to adapt to various environmental conditions and participate in beneficial plant-bacterial interactions. Beneficial *Bacillus* species can compete with plant-damaging microbes, inhibit plant pathogens, induce plant defense systems against pathogens, promote plant growth and increase nutrient uptake (Amallia *et al.* 2023). *Bacillus* species are commonly used as antagonistic bacteria of plant pathogens and plant growth promoters in industries and agriculture.

The potential of *Bacillus* for plant disease control is also supported by its ability to act in anti-quorum sensing by inhibiting the activity of *N*-acyl-homoserine (AHL) (Dong *et al.* 2002). *Bacillus* produces ACC deaminase, which can reduce plant ethylene content to increase plant resistance to environmental stresses (Onofre-Lemus *et al.* 2009) and phytase to hydrolyse organic phosphorus (Jorquera *et al.* 2011). *Bacillus* also synthesizes indole-3-acetic acid (IAA) to produce the hormone auxin (Raddadi *et al.* 2008) and glucose dehydrogenase to produce organic acids (Zubair *et al.* 2019). They can also dissolve phosphate (Suleman *et al.* 2018). Several studies have reported that *Bacillus* species effectively suppress *Colletrocrichum gloeosporioides* in chili, *Fusarium oxysporum* f.sp. *spinaciae* in spinach, *Phytophthora palmivora* in cocoa and *Rhizoctonia solani* in tomatoes (Ashwini & Srividya 2014; Arini *et al.* 2021; Szczech & Shoda 2006). Yanti *et al.* (2022) have reported that *Bacillus* species effectively improve shallot’s growth and productivity, also suppressing leaf blight disease caused by *Xanthomonas axonopodis* of shallot. Rahma *et al.* (2020) reported that *B. velezensis* B-27 promotes the growth and induces disease resistance of shallot. Likewise, *Bacillus*-treated shallots showed increasing induced resistance of twisted disease suppression by 72.2% to 100% (Wulan *et al*. 2022). In addition, Wibowo *et al*. (2022) revealed that the application of *Bacillus* species in off-season shallot production could enhance growth even though it could not inhibit *Fusarium* infection.

This study investigated the potential of *Bacillus* isolates for plant growth promotion and disease suppression on the basis of genes encoding plant-beneficial traits.

## MATERIALS AND METHODS

### Detection of Genes Encoding Plant-Beneficial Traits

This study used 18 *Bacillus* isolates from the collection of the Laboratory of Plant Pathology, Department of Plant Protection, Faculty of Agriculture, Universitas Gadjah Mada. These isolates were cultured on yeast peptone agar (YPA), which consists of 0.5% yeast extract, 1% polypeptone and 1.5% agar, and incubated for 48 h. DNA isolation was conducted using a Wizard® SV Genomic DNA Purification System kit (Promega, USA) following the protocol provided by the manufacturer (Prakoso *et al*. 2022). The obtained DNA was then amplified through the polymerase chain reaction (PCR) technique. The PCR products were analysed through a 1.2% agarose gel; the list of specific primers used in this study is shown in Table 1.

### Maize Seed Treatments

*Bacillus* isolates were cultured on YPA media to a density of 10^8^ CFU/mL. Maize seeds were washed with sterile water, and 10 seeds were drained with filter paper. The seeds were germinated in a Petri dish on filter paper moistened with sterile water. The germinated seeds were soaked in each bacterial suspension for 45 min. Seeds under the control treatment were soaked using sterile water and then grown in test tubes containing water agar (WA) media. The height, root length and fresh and dry shoot and root weights of each plant were recorded. Fresh and dry shoot and root weights were recorded two weeks after transfer to WA.

### *In vitro* Antagonistic Assay

The antagonism of *Bacillus* species against *Fusarium solani* (Lestiyani *et al*. 2016) was evaluated by coculturing both microorganisms side by side on the same potato dextrose agar (PDA) culture plate. The coculture assay was performed by preparing 10 mL of 0.6% WA media at 50°C and adding it to 100 μL of bacterial suspension with a density of 10^8^ CFU/mL. The mixed WA was then poured into solid PDA media in a Petri dish. A 0.5 cm × 0.5 cm agar culture disc of *F. solani* was then placed on top of the PDA plate and incubated for 7 days. Fungal growth was determined after 1 week by measuring the colony radius, which was compared with the colony radius of the control group (fungi grown on solid media and PBS solution in which bacteria had not been cultivated).

### Molecular Identification of Selected *Bacillus* Isolates

Selected *Bacillus* isolates that showed the best performance based on the genes encoding plant-beneficial traits, antagonism assay and maize seedling growth test were then amplified by PCR in accordance with Yamamoto and Harayama (1995) by using the primer pair gyrB-F (5′-CCC AAG CTT AAC TGC ACT GGG AAA TY-3′) and gyrB-R (5′-CGG AAT TCG GAT CCA CRT CGG CRT CB-3′) with the target amplicon size of ±1500 bp.

The PCR products amplified by using the *gyrB* primer were then submitted to 1st Base Company for sequencing analysis. The sequences were then analysed with BLASTn (https://blast.ncbi.nlm.nih.gov/Blast.cgi) to identify homologous samples by using the sequence data of several *Bacillus*-type strains obtained from GenBank. The sequence results were also deposited in GenBank to obtain accession numbers. The data were then aligned by applying MEGA X to construct a phylogenetic tree (Trianom *et al.* 2019).

### Field Experiments on Selected *Bacillus* Isolates

The field study was conducted with a completely randomised block design. The treatments tested were a combination of application methods (tuber dipping, plant spraying and tuber dipping plus plant spraying) using selected *Bacillus* isolates from previous experiments. The combination of treatments was as follows:

TD = tuber dipping using the *Bacillus* isolates with the highest number of plant growth-promoting genes and best growth performance in the seedling assay;
PS = plant spraying using the *Bacillus* isolates with the highest antagonistic activity;
DS = combination of tuber dipping using the *Bacillus* isolate with the highest number of plant growth-promoting genes and plant spraying using the *Bacillus* isolate with the highest antagonistic activity;
Control = no bacterial application.

The tuber dipping method involved dipping a shallot tuber in the selected *Bacillus* suspension with a density of 10^8^ CFU/mL for 30 min. The plant spraying method involved spraying the shallot 7 days after planting (dap) with the selected *Bacillus* suspension with a density of 10^8^ CFU/mL using a hand sprayer. Spraying was done once a week for 6 weeks.

### Field Observation of Plant Growth and Twisted Disease Intensity

Field observations were conducted once every two weeks for six weeks. The agronomic parameters observed were plant height and leaf number. Twisted disease intensity was determined using the scores of the symptomatic plants in each block.

The twisted disease symptom scores of shallots in the field were assessed by following: (Köycü and Özer 1997): Score 0 = no symptoms, score 1 = 1%–20% of the leaves yellowed and twisted, score 2 = 20%–40% of the leaves yellowed and twisted, score 3 = 41%–60% of the leaves yellowed and twisted, score 4 = 61%–80% of the leaves yellowed and twisted and score 5 = 81%–100% of the leaves yellowed and twisted. Disease intensity was calculated by using the following formula (Widyaningsih *et al.* 2017):


$$\text{Disease intensity}=\frac{\Sigma (n\text{i}\times v\text{i})}{Z\times N}\times 100\%$$

where *n* = number of infected plants having the same score, *v* = severity score, *Z* = maximum rating scale number and *N* = total number of plants observed.

The value of the area under the disease progress curve (AUDPC) was determined by using the formula as done by Widyaningsih *et al*. (2019):


$$\text{AUDPC}=\sum_{i}^{n-1}\left(\frac{Yi+Yi+1}{2}\right)\;(t_{i+1}-t_{i})$$

where *n* = total number of observations, *yi* = assessment of disease intensity at the *i*-th observation, *yi* + 1 = assessment of disease intensity at the *i*-th + 1 observation, *t_i_* = time at the *i*-th observation and *t_i_* + 1 = time at the *i*-th + 1 observation.

### Statistical Analysis

The data on plant height and leaf number were tested by using an analysis of variance with a confidence level of 95%. The significance of the differences was then further analysed using Duncan’s multiple range test at a confidence interval of 95%.

## RESULTS

### Detection of Genes Encoding Plant-Beneficial Traits

**Figure 1:**
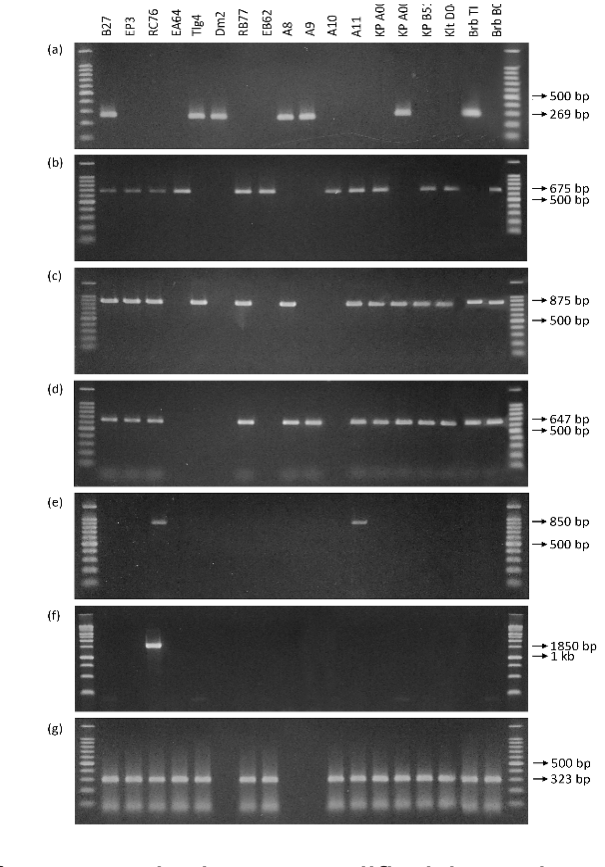
DNA bands of *Bacillus* isolates amplified by using specific primers for genes encoding (a) *fenD*, (b) *sfp*, (c) *bamC*, (d) *ituA*, (e) *aiiA*, (f) *ipdC* and (g) *nifH*. DNA bands were visualised on 1.2% agarose gel with a 1 kb DNA ladder. The presence of DNA bands indicates a positive result.

In this study, we used a pair of specific primers to detect each gene encoding antibiotics (fengycin, surfactin, bacyllomicin and iturin), indole pyruvate decarboxylase, acyl-homoserine lactonase and nitrogenase. The molecular detection results showed that among the 18 *Bacillus* isolates, seven, including B-27, Tlg4, Dm2, A8, A9, KP A004 and Brb T1, had the gene encoding fengycin (*fenD*) with the amplicon size of 269 bp (Fig. 1a). All isolates, except for Tlg, Dm2, A8, A9, KP A004 and Brb, had the gene encoding surfactin (*sfp*) with an amplicon size of 675 bp (Fig. 1b). All isolates, except for EA64, Dm2, EB62, A9 and A10, had the gene encoding bacyllomicin D (*bamC*), which had a DNA band with a size of 875 bp (Fig. 1c). All isolates, except for EA64, Tlg4, Dm2, EB62 and A10, had genes encoding iturin A (*ituA*) as indicated by the amplification of a DNA band with a size of 647 bp (Fig. 1d). Only isolate RC76 had the gene encoding indole pyruvate decarboxylase (*ipdC*) as indicated by the presence of a DNA band with a size of 1850 bp (Figure 1e). Two isolates (RC76 and A11) contained the gene encoding acyl-homoserine lactonase (*aiiA*) as illustrated by the amplification of a DNA band with a size of 850 bp (Fig. 1f). All isolates, except for Dm2, A8 and A9, had a gene encoding nitrogenase (*nifH*), as demonstrated by the amplification of a DNA band with a size of 323 bp (Fig. 1g).

### Screening of *Bacillus* Isolates on the Basis of Maize Seedling Growth

Screening was performed to identify isolates with a positive effect on the growth of maize seeds. The selected isolates were then applied in the field. The results showed that compared with the control treatment, isolates B27 and RC76 significantly affected plant growth. Treatment with isolates B27 and RC76 produced the highest plant heights of 22.80 cm and 22.33 cm, respectively (Table 2). Treatment with *Bacillus* species resulted in greater increases in plant height than the control treatment. Similarly, under treatment with isolate B27, the root length reached 19.10 cm.

Treatment with isolate RC76 resulted in the highest fresh weight of shoots and fresh weight of roots of 68 mg and 82 mg, respectively. Treatment with isolate B27 resulted in fresh crown and root weights of 64 mg and 88 mg, respectively. Treatment with the two isolates resulted in higher fresh shoot and root weights of 33 mg and 52 mg, respectively, than the control treatment (Table 2).

The application of PGPB affected the dry weight of maize seedlings. Under treatment with the RC76 isolate, the highest shoot and root dry weights were 5.8 mg and 11.4 mg, respectively, and treatment with isolate B-27 resulted in the highest shoot and root dry weights of 6.0 mg and 13.0 mg, respectively (Table 2).

### *In vitro* Antagonistic Assay

**Figure 2:**
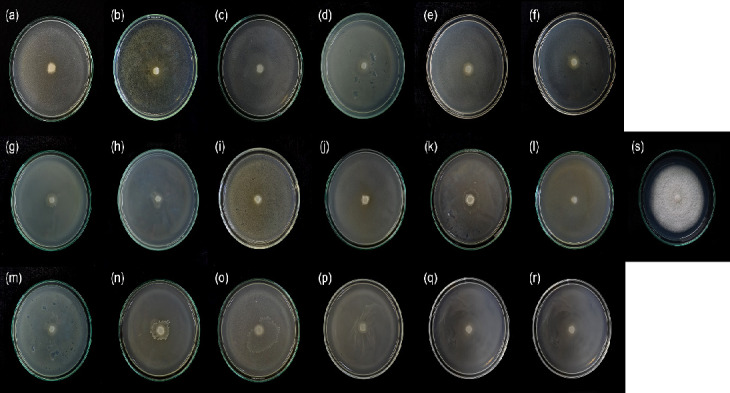
Assay on the antagonism of *Bacillus* isolates against *F. solani* on day 7 by coculture method. (a) B-27, (b) EP3, (c) RC76, (d) EA64, (e) Tlg4, (f) Dm2, (g) RB77, (h) EB62, (i) A8, (j) A9, (k) A10, (l) A11, (m) KP A003, (n) KP A004, (o) KP B51, (p) Klt D04, (q) Brb T1, (r) Brb B01 and (s) control.

The *in vitro* antagonistic assay results revealed that *Bacillus* isolates could suppress the growth of *F. solani*. The coculture technique was applied to investigate the influence of the 18 *Bacillus* isolates on the hyphal growth of *F. solani*. The *Bacillus* isolates significantly exhibited different inhibition rates against *F. solani* growth (Table 3 and Fig. 2). *Bacillus* isolate B-27 was significantly the most effective with an inhibition of 87.36%, followed by Dm2, A8 and KP B51, with an inhibition of 86.78%.

### Molecular Identification of Selected *Bacillus* Isolates

**Figure 3:**
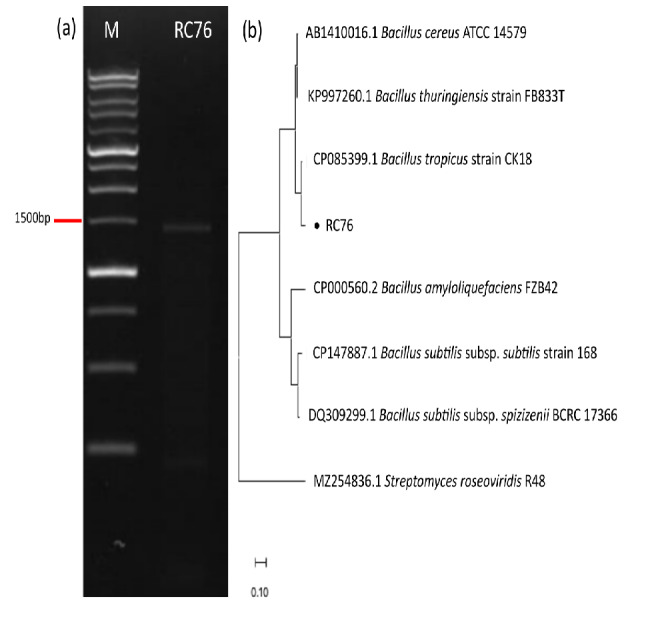
(a) gyrB gene of the *Bacillus* isolate RC76 on 1.2% agarose with an amplicon size of ±1,500 bp; (b) Phylogenetic tree analysis of the *Bacillus* isolate RC76.

B-27 and RC76 were further identified as having the highest potential as PGPB based on gene encoding detection and in vitro antagonistic assays. Rahma *et al.* (2020) found that isolate B27 had a high similarity (99%) with *B. velezensis* (MN905547). The molecular identification of isolate RC76 was conducted using a pair of universal primers gyrB-F/gyrB-R. An amplicon size of ±1500 bp was obtained. The appearance of the DNA band was observed on agarose gel 1.2% (Fig. 3a). BLAST revealed that the nucleotide sequence of the PCR product had the highest similarity with that of *B. tropicus* CK8. The nucleotide sequence data were deposited in GenBank with the accession number OL420681. Phylogenetic analysis utilising the neighbour-joining method with 1000 bootstraps revealed that isolate RC76 belonged to the same clade as *B. tropicus* and had the highest similarity of 99.96% with *B. tropicus* CK8 (Fig. 3b).

### Plant Growth and Twisted Disease Intensity of Shallot

**Figure 4:**
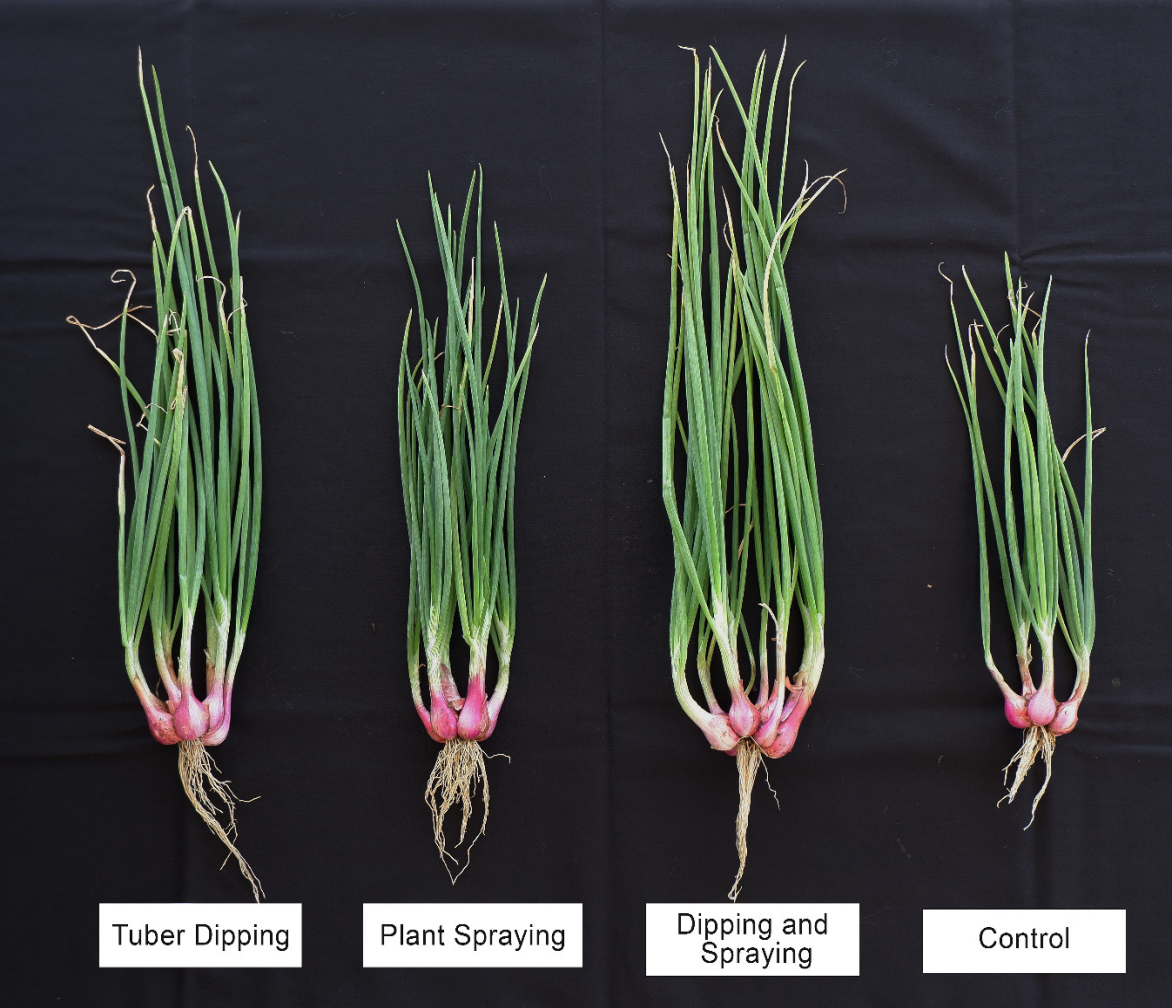
Effect of *Bacillus* treatments on shallot growth at six weeks after planting. *Notes.* TD = tuber dipping using B. tropicus RC76; PS = plant spraying using B. velezensis B-27; DS = tuber dipping with B. tropicus RC76 + plant spraying with B. velezensis B-27.

The efficiency of *B. tropicus* RC76 and *B. velezensis* B-27 in promoting plant growth and suppressing twisted disease in shallot was assessed in a field experiment. *Bacillus*-treated shallots showed better agronomic characteristics than the control plants (Fig. 4). Plant height and leaf number under treatment with *Bacillus* isolates significantly differed from those under other treatments at 32.1 cm and 21.73, respectively (Table 4). Combined treatments, including tuber dipping with *B. velezensis* B-27 suspension and plant spraying with *B. tropicus* RC76 suspension, showed a synergistic effect on plant growth promotion. The *Bacillus* supported these results, which had been molecularly detected as having plant growth-promoting genes such as *ipdC* and *nifH*.

*Bacillus*-treated shallot showed a significant result in suppressing twisted disease intensity than the control plot. The control plot showed the highest twisted disease intensity (25%), followed by the plot subjected to tuber dipping with *B. tropicus* RC76 (18%), the plot subjected to combined treatment with tuber dipping with *B. tropicus* RC76 and plant spraying with *B. velezensis* B-27 (11%) and the plot subjected to plant spraying with *B. velezensis* B-27 showed the highest effect on suppressing twisted disease intensity (9%) (Table 5). Applying *Bacillus* species in the field decreased twisted disease intensity in shallots by 42%–64%.

**Figure 5:**
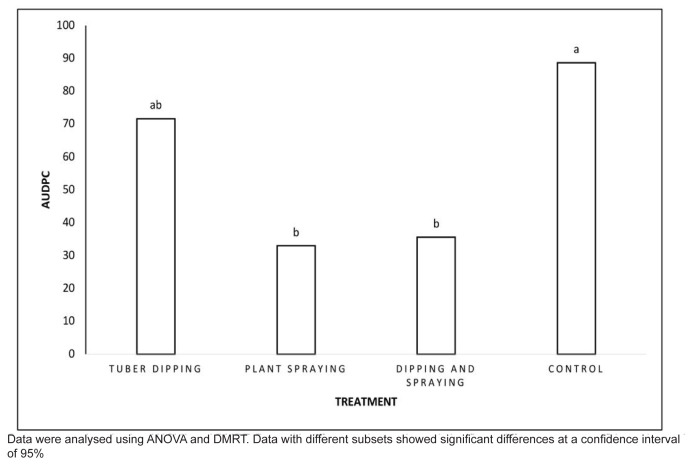
The area under disease progress curve (AUDPC) after six weeks of treatment with *B. velezensis* B-27 and *B. tropicus* RC67. Data were analysed using ANOVA and DMRT. Data with different subsets showed significant differences at a confidence interval of 95%

AUDPC analysis revealed a significant result, indicating that the development of twisted disease was highest in the control plot (88.67), followed by the plots treated with tuber dipping, dipping and spraying, and plant spraying (Fig. 5). These results corresponded to the twisted disease intensity in each plot. The low AUDPC indicated that the application of *Bacillus* species could inhibit the growth of *F. solani*.

## DISCUSSION

This study investigated the potential of *Bacillus* species as beneficial bacteria that could promote plant health by using 18 isolates collected from different hosts. Although there are many reports on plant growth-promoting bacteria, this study was not only carried out in the field but also in the laboratory to screen isolates by detecting the plant growth-promoting genes and *in vitro* antagonist assays, followed by testing the effectiveness of selected isolates in the field. Antibiotic synthesis by beneficial microorganisms is the most effective mechanism for controlling pathogens and improving plant health. This study detected and screened genes encoding beneficial traits in *Bacillus* isolates using PCR. These genes encoded surfactin, fengycin, bacillomycin and iturin. All *Bacillus* isolates used in this study could synthesise various antibiotics. Most of the isolates could produce surfactin, bacillomycin and iturin. This finding is supported by previous studies, which reported that some *Bacillus* species could produce three types of antibiotics (Ramarathnam *et al.* 2007). Surfactin, bacillomycin and iturin are the most common lipopeptide antibiotics produced by *Bacillus* species. Stanković *et al.* (2012) also detected several antibiotic-coding genes, including surfactin, fengycin, bacillomycin and iturin genes, in *Bacillus* species. *Bacillus amyloliquifaciens* Q-426 has several broad-spectrum antibiotic genes, such as genes encoding fengycin A, surfactin, iturin and bacillomycin D (Zhao *et al.* 2013). The production of surfactin, iturin and bacillomycin indicates strong antifungal activity, and surfactin has the strongest biosurfactant ability. The active compound on the surface of surfactin plays a role in bacterial development because it participates in biofilm formation (Hofemeister *et al.* 2004). Iturin has limited antibacterial activity but a wide range of antifungal activities (Ye *et al.* 2012). Besides having a strong antibiotic function, iturin could increase the swarming motility of bacteria (Alina *et al.* 2015; Joko *et al*. 2007). Roongsawang *et al.* (2002) reported that *Bacillus amyloliquefaciens*, *B. licheniformis*, *B. pumulis* and *B. subtilis* could produce iturin. Bacillomycin D produced by *B. amyloliquefaciens* FZB42 was documented to induce morphological changes in the plasma membrane and hyphal and conidial cell walls of *Fusarium graminearum*, thus leading to cell death (Gu *et al.* 2017). Fengycin also affects cell membranes and organelles, inhibiting DNA synthesis and decreasing virulence levels in *F. graminearum* (Hanif *et al.* 2019). Fengycin inhibits biofilm formation by several gram-negative bacteria and induces ISR. *Bacillus* species that could produce fengycin include *B. subtilis*, *B. licheniformis* and *B. amyloliquefaciens* (Alina *et al.* 2015).

In this study, two *Bacillus* species isolates, namely RC76 and A11, were found to contain AHL lactonase (*aiiA*) coding genes. AHL lactonase is an enzyme that degrades *N*-AHL, a quorum-sensing signalling molecule in Gram-negative bacteria. AHL lactonase can hydrolyse quorum-sensing signal molecules and constrain bacterial communication systems. In this case, microorganisms producing AHL lactonase have the potential to be used as biological control. Dong *et al.* (2000) reported that AHL lactonase, first identified in *Bacillus* species, can inactivate bacterial pathogenicity via quorum-sensing through the hydrolysis of the AHL lactone ring. Dong *et al.* (2002) discovered three *Bacillus* species, namely, *Bacillus thuringiensis*, *B. cereus* and *B. mycoides*, with AHL lactonase-coding genes.

The detection of antibiotic genes in *Bacillus* species in this study showed that antibiosis is a mechanism involved in biological control. All isolates used in this study inhibited the mycelial growth of *F. solani* by more than 65% *in vitro*. Among the isolates tested, *B. velezensis* B-27 showed the greatest antibiotic role in biocontrol, as evidenced by its ability to inhibit the mycelial growth of *F. solani* by 87.36%. This isolate was found to produce several antibiotics, such as fengycin, surfactin, bacillomycin and iturin. This result is supported by Zhao *et al.* (2013), who reported that fengycin D produced by *B. amyloliquifaciens* Q-426 could inhibit spore germination and suppress the mycelial growth of *F. oxysporum* f.sp. *spinaciae* O-27. Ramarathnam *et al.* (2007) also reported that *B. subtilis* DFH09 significantly inhibited the mycelial growth of *F. graminearum* by 60%. Jayanti and Joko (2020) also documented that *Bacillus* isolates could inhibit the mycelial growth of *F. oxysporum* f.sp. *melonis.*

This study also discovered that *Bacillus* species could produce phytohormones, such as the auxin hormone IAA, thus supporting plant health. *Bacillus tropicus* RC76 is the only isolate in which the *ipdC* gene was detected. This gene is responsible for IAA synthesis via the indole pyruvate pathway. Other isolates could likely also produce IAA via the indoleacetic acid pathway. Raddadi *et al.* (2008) reported that *B. thuringiensis* possesses the *ipdC* gene. Dwimartina *et al.* (2017) also reported that *B. cereus* could produce IAA, as indicated by a colour change in Salkowski’s reagent. Tsavkelova *et al.* (2007) explained that IAA production by microorganisms could stimulate root formation and plant growth.

The potential of *Bacillus* species as PGPB is also supported by their ability to fix nitrogen. In this study, most isolates carried the *nifH* gene, which encodes nitrogenase. This result indicated that the *nifH* gene is common among *Bacillus* species. Several *Bacillus* species that have been reported to carry the *nifH* gene include *Bacillus megarerium*, *B. cereus*, *Bacillus firmus*, *B. pumulis*, *B. subtilis*, *Bacillus marisflavi*, *B. licheniformis*, *Bacillus circulans* and *Bacillus oceanisediminis* (Xiu *et al.* 2006; Yousuf *et al.* 2017). In general, *nifH* is required for nitrogen fixation, is involved in iron protein activation and iron cofactor biosynthesis, and is a regulatory gene needed for the synthesis and function of enzymes (Souza *et al.* 2015).

Based on laboratory experiments, *B. velezensis* B-27 and *B. tropicus* RC76 were selected for field experiments because they have potential traits for improving plant health. *B. tropicus* RC76 has the most favourable traits, including genes for surfactin, bacillomycin, iturin, AiiA, nitrogenase and IAA, and is suitable for application via tuber dipping. *B. velezensis* B-27 has the potential to control pathogens given that it possesses genes for fengycin, surfactin, bacillomycin, iturin and nitrogenase and is suitable for application through plant spraying. The field experiment on efficacy showed that *Bacillus* isolates applied individually and in combination resulted in higher plant height and leaf number than the control treatment. Patten and Glick (1996) stated that the ability to produce auxin is the most widely reported mechanism underlying the role of PGPB in plant growth. Approximately 80% of rhizosphere microbes can synthesise and release auxin as a secondary metabolite. According to Jorquera *et al.* (2014), nitrogen-fixing bacteria can be used as nitrogen fertiliser to increase plant growth. The findings of this research are in line with the study by De la Vega-Camarillo *et al.* (2023), who found that PGPB can increase the plant height, root dry weight, root length and number of leaf maize seedlings *in vitro* and produce secondary metabolites that act as antibiotics.

*Bacillus* species that are PGPB have the ability to stimulate plant growth and suppress disease development through direct and indirect mechanisms because they can produce the auxin hormone IAA and the enzyme phosphomonoesterase (Castaldi *et al*. 2021). Given that IAA actively promotes cell development and stimulates the formation of new roots, and can spur growth and increase enzyme activity, the addition of PGPB can enhance the quality of plant growth and yield (Ilmiah *et al.* 2021; Ratnaningsih *et al.* 2023). Rahnama *et al.* (2023) also demonstrated the effect of PGPB *Bacillus* species on plant growth. They reported that the application of PGPB to plants can reduce the use of chemical fertilisers, pesticides and hormones used for plant growth and can increase plant height, root length and plant dry weight. In addition to increasing plant height and triggering root elongation, *Bacillus* species can increase the fresh and dry weights of plants. Elemental phosphorus has the benefit of stimulating root growth, particularly the roots in young plants. Root formation increases water and nutrient uptake. The increase in fresh weight is caused by the uptake of a sufficient amount of water by plant cells and increases photosynthesis (Abdullah *et al.* 2024). *Bacillus* species also act as PGPB through their ability to fix N_2_ from the air and convert nitrogen into NO_3_^−^ for plants, thus helping provide nitrogen elements and minimising nitrogen loss to meet plant growth (Rahnama *et al*. 2023). Plants need elemental nitrogen because the development of their tissues is largely determined by the availability of nitrogen elements, which participate in the rapid formation of vegetative parts and the generative phase through cell division, cell elongation and enlargement in meristem tissues that then form new cell walls and protoplasm (Muratore *et al*. 2021).

The data obtained in this work showed that maize plants treated with biological agents had higher dry weights than those that received the control treatment. Katsenios *et al.* (2022) showed that in maize, inoculating seeds with PGPB can increase plant dry weight and germination at low temperatures because the application of biological agents can increase cell development, stimulate new root formation, spur growth, stimulate flowering and increase enzyme activity as a function of the hormone auxin. The hormone auxin and the enzyme nitrogenase increase the dry weight and nutrient uptake of maize plants (Sosnowski *et al*. 2023). The screening of 18 *Bacillus* isolates identified B27 and RC76 as isolates that triggered higher plant growth than other isolates.

The application of *B. velezensis* B-27 isolate on shallots through plant spraying provided the best result in suppressing the development of twisted disease in the field. The low intensity of twisted disease was most likely influenced by the metabolites produced by *B. velezensis* B-27. These metabolites include the antifungals fengycin, surfactin, bacyllomicin D and iturin A. These results are in accordance with the findings of Rahma *et al.* (2020), who reported that *B. velezensis* B27 reduced the intensity of twisted disease in shallots by up to 67%. Athukorala *et al.* (2009) reported that *B. cereus* L-01-07 produced surfactin antibiotics, iturin A, bacyllomicin D and zwittermicin A and inhibited the growth of *F. graminearum* by 52% (Ramarathnam *et al.* 2007). Athukorala *et al.* (2009) also identified several strains of *B. amyloliquifaciens* that can produce surfactin antibiotics, iturin A, bacyllomicin D and zwittermicin A. Farzand *et al.* (2019) reported that *B. amyloliquifaciens* can suppress *Sclerotinia sclerotium* infection in the leaves and stems of canola. In addition, Wang *et al.* (2019) reported that *B. cereus* AR156 could suppress bacterial wilt disease caused by *Ralstonia solanacearum* by 51.02%. The formation of a biofilm around the root surface and the secretion of antibiotic toxins (surfactin, fengycin, bacyllomicin and iturin) by *Bacillus* species interfere with the development of pathogenic fungal populations and reduce the incidence of disease in plants. The secretions of *Bacillus* species kill pathogenic fungi by degrading cell walls and changing cell morphologies (Radhakrishnan *et al.* 2017).

## CONCLUSION

The two selected *Bacillus* species isolate with the best potential to improve shallot plant health are *B. velezensis* B-27 and *B. tropicus* RC76. Gene detection revealed that *B. velezensis* B27 had several genes encoding antifungals, including fengycin, surfactin, bacyllomicin and iturin, and a gene encoding nitrogenase. *B. tropicus* RC76 possessed antifungal genes, including surfactin, bacyllomicin, iturin, nitrogenase and quorum quenching genes. Applying *B. velezensis* B-27 and *B. tropicus* RC76 increased plant height and leaf number. The application of *B. velezensis* B-27 could suppress disease development.

## Figures and Tables

**Table 1:** List of primers for genes encoding plant-beneficial traits in *Bacillus* species.

Translation product	Gene	Primer	Primer sequence (5′-3′)	Annealing (°C)	Reference
*Antiquorum sensing*	*aiiA*	aiiA_240B1_ aiiA_COT1_	ATGGGATCCATGACGTAAAGAAGCTTTAT GTCGAATTCCTCAACAAGATACTCCTAATG	55	Dong *et al*. (2002)
Fengycin	*fenD*	FENDF FENDR	GGCCCGTTCTCTAAATCCAT GTCATGCTGACGAGAGCAAA	62	Mora *et al*. (2011)
Bacyllomicin D	*bamC*	BACC1F BACC1R	GAAGGACACGGCAGAGAGTC CGCTGATGACTGTTCATGCT	60	Ramarathnam *et al*. (2007)
Iturin A	*ituA*	ITUD1F ITUD1F	GATGCGATCTCCTTGGATGT ATCGTCATGTGCTGCTTGAG	60	Athukorala *et al*. (2009)
Surfactin	*sfp*	P17 P18	ATGAAGATTTACGGAATTTA TTATAAAAGCTCTTCGTACG	46	Hsieh *et al*. (2004)
Indolepyruvate decarboxylase	*ipdC*	F-idpC R-idpC	CAYTTGAAAACKCAMTATACTG AAGAATTTGYWKGCCGAATCT	50	Raddadi *et al*. (2008)
Nitrogenase	*nifH*	nifH-F nifH-R	GCTGCGATCCVAAGGCCGAYTCVACCCG CTGVGCCTTGTTYTCGCGGATSGGCATGGC	55	Ding *et al*. (2005)

**Table 2:** Effects of the addition of *Bacillus* species on the growth of maize seedlings inoculated with *Bacillus* isolates.

Isolates	Plant height (cm)	Root length (cm)	Fresh weight (mg)		Dry weight (mg)	

			Shoot	Root	Shoot	Root
B27	22.80 ^a^	19.10 ^a^	64 ^a^	88 ^a^	6.0 ^a^	13.0 ^a^
RC76	22.33 ^a^	16.28 ^abc^	68 ^a^	82 ^ab^	5.8 ^ab^	11.4 ^ab^
RB77	21.16 ^ab^	18.04 ^a^	55 ^abc^	82 ^ab^	5.0 ^ab^	8.0 ^bc^
Brb B01	21.14 ^ab^	12.86 ^ab^	54 ^abc^	64 ^abcde^	5.0 ^ab^	9.0 ^abc^
A4	20.79 ^ab^	15.28 ^abcde^	47 ^abc^	58 ^cde^	3.6 ^abcd^	6.4 ^c^
KP A003	20.30 ^ab^	16.18 ^abc^	61 ^ab^	70 ^abcde^	5.2 ^ab^	8.4 ^abc^
Klt D04	20.28 ^abc^	15.18 ^abcde^	60 ^ab^	68 ^abcde^	4.8 ^ab^	8.2 ^abc^
KP A004	20.04 ^abc^	12.46 ^cde^	64 ^ab^	67 ^abcde^	6.0 ^a^	8.0 ^bc^
EA64	19.58 ^abc^	14.94 ^abcde^	53 ^abc^	59 ^bcde^	4.6 ^ab^	7.4 ^bc^
EP3	19.40 ^abc^	17.78 ^ab^	55 ^abc^	79 ^abc^	4.0 ^abc^	9.0 ^abc^
Tlg4	18.90 ^abc^	15.40 ^abcd^	60 ^ab^	74 ^abcd^	4.2 ^ab^	9.0 ^abc^
EB62	18.80 ^abc^	11.10 ^e^	45 ^abc^	61 ^bcde^	3.0 ^e^	8.0 ^bc^
Dm2	17.90 ^abc^	14.84 ^cde^	60 ^ab^	66 ^abcde^	4.6 ^ab^	9.2 ^ab^
A11	17.90 ^abc^	18.08 ^a^	50 ^ab^	74 ^abcd^	4.0 ^abc^	9.0 ^abc^
KPB51	17.70 ^abc^	15.66 ^abcd^	57 ^ab^	57 ^de^	4.2 ^ab^	9.2 ^ab^
A8	17.62 ^abc^	12.92 ^cde^	41 ^abcd^	64 ^abcde^	3.0 e	7.0 ^bc^
A10	17.40 ^abc^	15.02 ^abcde^	46 ^abc^	65 ^abcde^	4.2 ab	7.6 ^bc^
Brb T1	14.52 ^bc^	11.64 ^de^	38 ^abcd^	57 ^de^	3.0 e	8.0 ^bc^
Control	11.00 ^c^	8.62 ^e^	33 ^e^	52 e	3.0 ^e^	6.0 ^c^

^*^Note. Values followed by the same letters in the same column are not significantly different.

**Table 3:** Antagonistic activity of 18 isolates of *Bacillus* species on the growth of *Fusarium solani* on day seven after testing.

Isolates	Inhibition (%)	Isolates	Inhibition (%)
B-27	87.36 ^a^	A9	86.21 ^ab^
EP3	86.21 ^ab^	A10	72.99 ^e^
RC76	77.01 ^d^	A11	58.62 ^e^
EA64	82.76 ^c^	KP A003	82.76 ^c^
Tlg4	83.91 ^bc^	KP A004	74.14 ^e^
Dm2	86.78 ^a^	KP B51	86.78 ^a^
RB77	82.76 ^c^	Klt D04	81.03 ^c^
EB62	85.63 ^ab^	Brb T1	65.52 ^e^
A8	86.78 ^a^	Brb B01	66.67 ^e^

*Notes.* Data were analysed using ANOVA and DMRT. Data with different subsets showed significant differences at a 95% confidence interval.

**Table 4:** Effect of *Bacillus* treatments on shallot growth four weeks after planting.

Treatment	Plant height (cm)	The number of leaves
TD	28.53 ^ab^	20.48 ^ab^
PS	29.89 ^ab^	18.95b ^c^
DS	32.10 ^a^	21.73 ^a^
Control	26.95 ^b^	17.32 ^c^

*Notes.* TD = tuber dipping; PS = plant spraying; DS = dipping and spraying. Data were analysed by using analysis of variance and Duncan’s multiple range test. Data with different subsets showed significant differences at a 95% confidence interval.

**Table 5:** Effect of *Bacillus* species treatments on twisted disease intensity in shallots at six weeks after planting.

Treatment	Disease intensity (%)
TD	18 ^ab^
PS	9 ^b^
DS	11 ^b^
Control	25 ^a^

*Notes.* TD = tuber dipping; PS = plant spraying; DS = dipping and spraying. Data were analysed using ANOVA and DMRT. Data with different subsets showed significant differences at a 95% confidence interval.
